# Effect of Everyday Life Rehabilitation on recovering quality of life in individuals with serious mental illness in supported accommodation: a pragmatic cluster randomised controlled trial

**DOI:** 10.1136/bmjment-2025-301757

**Published:** 2025-08-07

**Authors:** André Sjöberg, Per Liv, Maria Lindström

**Affiliations:** 1 Department of Epidemiology and Global Health, Umeå University, Umeå, Västerbotten County, Sweden; 2 Department of Public Health and Clinical Medicine, Umeå University, Umeå, Västerbotten County, Sweden

**Keywords:** Adult psychiatry, Schizophrenia & psychotic disorders

## Abstract

**Background:**

Individuals with serious mental illness (SMI) living in supported accommodation often lead lonely and sedentary lives. Everyday Life Rehabilitation (ELR) is a collaborative, person-centred, activity-oriented and recovery-oriented intervention that integrates outreach rehabilitation efforts into routine practices. This intervention aims to enhance personal recovery and quality of life by promoting engagement in meaningful everyday activities within real-life contexts.

**Objective:**

To evaluate the effectiveness of ELR on personal recovery and quality of life among residents with SMI in supported accommodation, compared with treatment-as-usual (TAU).

**Methods:**

This was a pragmatic, parallel-group, cluster-randomised controlled trial (RCT) (NCT05056415) conducted in Sweden between August 2021 and June 2024. The RCT included 60 housing units (clusters) randomly assigned (1:1) to receive either ELR or TAU. Data were collected by independent, blinded assessors, with partial blinding of residents. The primary outcome, Recovering Quality of Life (ReQoL-20), was assessed at the individual level and analysed using a mixed-effects model and an intention-to-treat (ITT) approach by a statistician blinded to the allocation.

**Findings:**

Participants in the intervention group showed significantly greater improvements in ReQoL scores at 6 months compared with the control group (20.1, 95% CI: 15.8 to 24.4), with a statistically significant between-group difference (p<0.001). The ITT analysis included 60 housing units with 161 participants (86 men and 72 women), of whom 90 were allocated to ELR (33 units) and 71 to TAU (27 units). The overall attrition rate was 24% in both groups, and no major adverse events were reported.

**Conclusions:**

These findings indicate that ELR is an effective intervention with a clinically relevant impact on recovering quality of life for individuals with SMI living in supported accommodation. While these results should be interpreted within the context of the Swedish system, they contribute to the growing body of evidence supporting recovery-oriented and activity-oriented interventions in supported accommodation.

**Clinical implications:**

Responsive, person-centred, goal-oriented activity training, grounded in collaborative alliance, represents a valid strategy for recovery-oriented interventions. While multilevel approaches must be tailored to specific contexts, the integration of occupational therapists may provide clinical benefits in supported accommodation.

**Trial registration number:**

NCT05056415.

**WHAT IS ALREADY KNOWN ON THIS TOPIC**

- Recovery-oriented interventions are a policy priority in many countries, but their effectiveness, particularly those relying on staff education, remains inconclusive.
- Residents and staff in supported accommodation often lack access to tailored, evidence-based interventions that meet their specific needs.

**WHAT THIS STUDY ADDS**

- This trial shows that Everyday Life Rehabilitation significantly improves recovering quality of life for individuals with serious mental illness in supported accommodation, demonstrating the rehabilitative value of meaningful everyday activities in promoting both engagement and personal recovery.

**HOW THIS STUDY MIGHT AFFECT RESEARCH, PRACTICE OR POLICY**

- Policymakers and providers should prioritise context-specific, multilevel interventions in supported accommodation, recognising activity engagement and personal recovery as both outcomes and mechanisms of change.
- Gradual, real-world activity training, supported by the CHIME principles (Connectedness, Hope, Identity, Meaning and Empowerment), and an occupational therapist, in collaboration with the resident and housing staff, may provide clinical benefits when used in supported accommodation.

## Background

 ’Supported accommodation’ is a common community-based service for individuals with serious mental illness (SMI).^1^ In Sweden, supported accommodation alternatives vary but generally follow a no-move-on approach, including traditional residential care homes reserved for individuals with extensive and complex needs.^2^ Although these services were developed to be less restrictive than older psychiatric institutions, they unintentionally replicate many of their flaws.^3 4^ Residents with SMI often experience loneliness and a sedentary lifestyle due to limited opportunities for meaningful activities and the challenges associated with their conditions.^3 5^ Additionally, the structured living environment and the high staff dependency in supported accommodation can make it challenging for residents to maintain a sense of agency, resulting in low self-determination and limited influence over everyday decisions.^6^,^8^

Recovery-oriented interventions have gained significant attention and policy recognition in recent decades.^9^ These approaches target personal recovery, often conceptualised by the CHIME framework (Connectedness, Hope, Identity, Meaning and Empowerment), emphasising the potential for individuals to lead meaningful lives despite disabilities and symptoms.^10^ Recovery-oriented interventions such as REFOCUS,^11^ PromQUAL (Promoting quality of care in residential units for people with long-term mental illness)^12^ and CARe (Comprehensive Approach to Rehabilitation)^13^ have introduced educational packages in order to improve staff interactions with residents. However, evidence regarding their effectiveness remains inconclusive, and educational packages alone seem to have limited impact on residents’ quality of life.^12 14^

Everyday Life Rehabilitation (ELR) is a collaborative, activity-oriented and recovery-oriented intervention that integrates personalised outreach rehabilitation efforts into the routine practices of supported accommodation.^15^,^17^ ELR does not rely solely on staff training but is a comprehensive, person-centred approach that fosters collaboration among occupational therapists, housing staff and residents, engaging them in meaningful everyday activities within real-life contexts. Engagement in meaningful activities is a key component of recovery,^18^ but housing staff often lack the resources, competencies and conditions needed to effectively integrate such activities into their daily practice.^4^ Although feasibility studies on ELR have shown promising results,^15^ there is a lack of controlled trials evaluating its effectiveness.

## Objective

This study aims to evaluate the effectiveness of ELR in promoting recovering quality of life among residents in supported accommodation, compared with treatment-as-usual (TAU). This study will estimate the intervention’s impact and test the hypothesis that personal recovery can be enhanced by targeting meaningful everyday activities. ELR is implemented at the housing unit level; therefore, a cluster-randomised design is used to ensure consistent application of the intervention within each unit while minimising contamination between the groups.

## Methods

### Study design

This is a pragmatic, parallel-group cluster randomised controlled trial (RCT) evaluating the effectiveness of ELR on recovering quality of life compared with TAU after 6 months for individuals with SMI living in supported accommodation, as detailed in the published study protocol.^16^ The project was organised into four waves to improve its practicality, with data collected from 60 housing units across Sweden between August 2021 and June 2024. Each wave, a new group of municipalities was recruited to participate in the RCT. The first two waves served as an internal pilot phase, allowing for necessary adaptations before proceeding with the full-scale RCT (Sjöberg *et al*, submitted 2024). Following the internal pilot, several changes were made to the study protocol, including the addition of a fourth wave and slightly expanded geographical area. These adaptations are detailed in online supplemental Appendix 1. The study design and statistical analysis plan (SAP) were registered on ClinicalTrials.gov (NCT05056415, registered on 4 September 2021) prior to participant enrolment. Reporting followed the CONSORT (Consolidated Standards of Reporting Trials) guidelines.

### Participants and recruitment

Initially, all municipalities within a 270 km radius of the city of Umeå were eligible to participate in the study. After the internal pilot, we expanded the eligibility criteria to include municipalities from Northern and Central Sweden to ensure an adequate sample size. Before randomisation, participating municipalities submitted lists of eligible units with residents diagnosed with SMI and sufficient access to occupational therapists. In this study, supported accommodation refers to services offering high levels of support without a move-on approach,^2^ typically involving community living arrangements with staff available around the clock. In Sweden, access to these accommodations is reserved for individuals with complex and severe disabilities. While this group generally includes those diagnosed with SMI, some residents may have multiple diagnoses, severe neuropsychiatric disorders or dual diagnoses involving substance use. Individuals with intellectual disabilities are covered by a separate legal framework and are not part of this study.

Potential participants were recruited through brochures distributed by housing staff or managers. While the brochure did not mention the presence of a control, they stated that it could take up to 6 months for them to receive ELR. These brochures included accessible information about the trial and intervention to help participants make an informed decision. Written informed consent was obtained from participants to ensure voluntary participation and protect their rights and confidentiality. The consent process was facilitated by external assessors trained in Good Clinical Practice (GCP) and experienced working with individuals with SMI, helping participants understand the study and its implications. All individuals who provided consent and met the inclusion criteria were enrolled. Eligible participants were adults (over 18 years) with an SMI diagnosis. Exclusion criteria’s was dementia, severe developmental disabilities, inability to communicate in Swedish, acute psychosis or acute suicide risk.

### Randomisation and masking

Randomisation was performed at the housing-unit level. The allocation was computer-generated by an external statistician (HH) using a 1:1 ratio, assigning participants to either ELR+TAU or TAU alone, stratified by municipality. Municipalities with only one participating housing unit were allocated to the intervention arm. Occupational therapists were centrally organised within municipalities and could be responsible for multiple housing units across both study groups, meaning that they were not randomised.

The enrolment and consent process was conducted by blinded assessors trained in GCP and standard procedures for data collection and management. Due to the nature of the intervention, allocation could not be concealed from occupational therapists, housing staff or managers. Residents were blinded to their study arm at baseline and were not informed of their allocation. However, once informed of their start date, residents may have deduced their study arm, potentially compromising blinding. Coded data were managed and stored by two university administrators (UN and KJ), who were not involved in the intervention or analysis. Data remained blinded to researchers until all analyses were completed and were stored in a locked, fire-safe box. The trial statistician (PL) remained blinded during the final analysis.

### Procedures

#### Intervention

**Figure 1:**
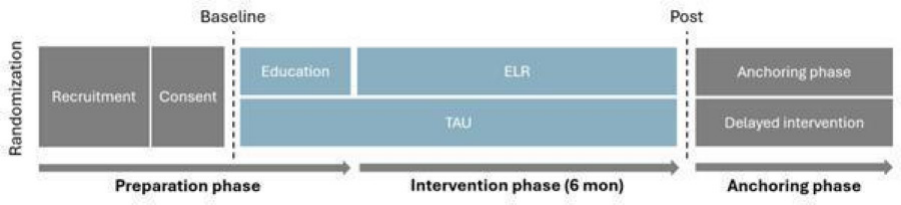
Study timeline. ELR, everyday life rehabilitation; TAU, treatment-as-usual.

ELR is designed to promote personal recovery through meaningful everyday activities for individuals with SMI living in supported accommodation. It provides individually tailored support to help residents work toward long-term recovery by encouraging their own will, motivation and hope. This is facilitated through exploration and training of everyday life activities and the achievement of personal goals. ELR is an integrated, collaborative model that involves residents, housing staff, occupational therapists and managers. The process begins with a preparation phase (figure 1) where housing staff distribute informational brochures to residents, encouraging them to start reflecting on their aspirations, before meeting with the occupational therapist. Before the intervention starts, the housing staff and occupational therapist complete a brief web-based educational package (10 prerecorded sessions, ∼3.5 hours), which familiarises them with person-centred, activity and recovery-oriented practices and the intervention manual. By completing similar training and applying tools for collaboration, both occupational therapists and housing staff establish a common language and consensus which can enhance intersectoral collaboration.

**Figure 2:**
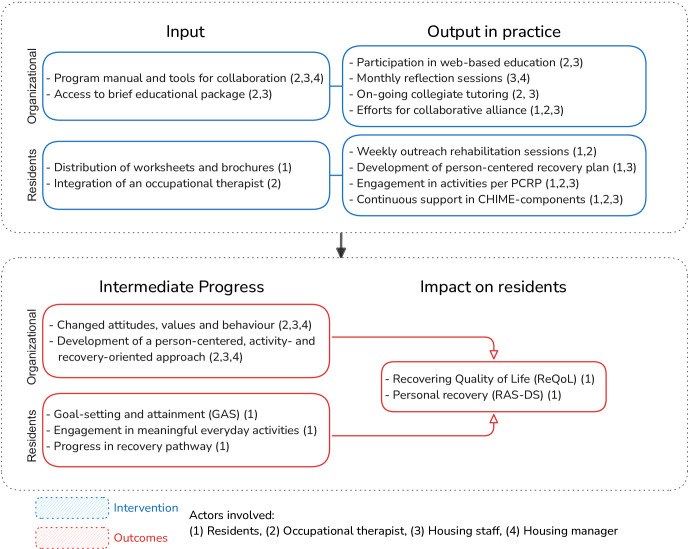
Illustration of the logic model. PCRP, person-centred recovery plan; CHIME, Connectedness, Hope, Identity, Meaning and Empowerment; RAS-DS, Recovery Assessment Scale-Domains and Stages.

The intervention phase involves weekly individual rehabilitation sessions between the resident and an occupational therapist. During the initial meetings, the residents’ interests, own will and activity priorities are collaboratively explored using a person-centred mapping approach. Through this shared decision-making process, a person-centred recovery plan (PCRP) is developed, outlining the goals, planned activities and strategies for the coming months of activity training in real-life context. Depending on the content of the PCRP, either the occupational therapist or the housing staff integrate the strategies of the PCRP into their daily practices. To engage the manager in supporting collegial learning among the housing staff, monthly reflection sessions took place following a structured format inspired by practice leadership.^19^ During these sessions, the manager was provided with a set of predefined discussion questions to guide the conversation. After 6 months, residents who have achieved their initial goals enter the anchoring phase, during which the occupational therapist and the resident evaluate further rehabilitation needs and plan how to maintain progress. Residents are encouraged to continue their activities with the support of housing staff. For more information on the intervention’s components and theoretical framework, see figure 2 or previous publications on ELR.^17 20^

#### Treatment-as-usual

In this pragmatic study, the units in the control group continued their current practices without receiving any standardised instructions, other than distributing informational brochures to residents. Current practices include a wide range of residential services and psychosocial support provided by housing staff, which vary depending on individual approaches, staff commitment and unit norms. Managers are typically based off-site with substantial workloads, placing considerable responsibility on housing staff to manage their daily practices.^21^ Co-planning for long-term rehabilitation is limited, and collaboration between occupational therapists and housing staff is often challenging. As a result, rehabilitation efforts tend to be short-term and sporadic, such as prescription of technical aids. To ensure fairness, a wait-list design was used, ensuring that the control group would receive access to the intervention after the study period.

### Outcomes and data collection

At baseline, residents completed a demographic information sheet and questionnaires for two patient-reported outcome measures (PROMs): the Recovering Quality of Life (ReQoL-20)^22^ and the Recovery Assessment Scale-Domains and Stages (RAS-DS).^23^ After 6 months, residents completed endpoint measurements for both the ReQoL-20 and RAS-DS. Both predata and postdata collection were facilitated by trained, blinded assessors who provided guidance throughout the process.

The primary outcome of this study was self-reported recovering quality of life after 6 months, measured using the 20-item version of the ReQoL-20. This is a concise and reliable tool for assessing personal recovery and quality of life in individuals with mental health conditions.^22^ It covers seven domains: meaningful activity, relationships, autonomy, hope, self-perception, well-being and physical health. Scores range from 0 to 80, with 0 representing the lowest quality of life and 80 the highest. The questionnaire has also been translated and linguistically validated in Swedish.^24^

The secondary outcome was self-reported recovery and daily functioning at 6 months, assessed using the Swedish translation of the RAS-DS instrument. This is a valid and reliable self-report measure of service user-defined recovery.^23^ It consists of 38 items rated on a Likert scale from ‘untrue’ to ‘completely true’ and covers four domains: valuing activities, looking forward, mastering illness and social belonging.

During the trial, the occupational therapist kept a written process protocol to monitor residents’ goals, activities and adherence. These protocols were used clinically in the intervention and are therefore only available for the intervention group. For those who received ELR, goal attainment was assessed by the occupational therapist using the Goal Attainment Scale (GAS), a criterion-referenced tool that measures progress on a 5-point scale (−2 to +2), with positive scores indicating better progress than expected.^25^ This assessment was submitted with the written process protocol. The protocols were also used to track adherence to the weekly sessions. The number of cancelled sessions was divided by the trial’s duration to calculate the overall adherence rate. To account for national holidays, 2 weeks were assumed to be cancelled and subtracted from the total duration for a more accurate adherence calculation.

### Sample size

The study was originally designed to detect a 5-point difference on ReQoL. However, following a non-comparative interim analysis and simulations based on the internal pilot, we redimensioned the study to be able to detect a 10-point difference (Sjöberg *et al*, submitted 2024). This adjustment aligns with the suggested minimum important difference for ReQoL-20.^26^ Considering the number of participants in wave three and four and the attrition rate during the first two waves, we estimated that the study would achieve 80% power to detect a clinically meaningful difference of 8.8 points on the ReQoL scale with 45 housing units. The SAP was updated accordingly. The interim analysis was performed by an independent and blinded statistician (SV), otherwise not involved in the study. Since the interim analysis did not compare outcomes between groups, there was no need to control the error rate in the final analysis. No information regarding group differences was shared to the research team. See online supplemental Appendix 1 for more details on the sample size recalculation.

### Statistical analysis

The primary analysis uses a modified intention-to-treat (ITT) approach, excluding municipalities and units that withdrew from the trial prior to the recruitment of residents. Missing data were handled using multiple imputations by chained equations (MICE). For ReQoL, participants with two or fewer missing values had values imputed using the mean of completed items, following the ReQoL scoring manual.^26^ The remaining missing values were imputed using partial mean matching from 10 possible donors, generating 30 imputed data sets. For RAS-DS, we calculated the mean for each domain and used these as predictors in MICE to impute the total RAS score.

Group differences in ReQoL were estimated using a mixed-effect model, with the 6-month follow-up measurement as the outcome and unit as random effect to account for the cluster design. Fixed effects included the intervention group and baseline ReQoL. Baseline ReQoL was adjusted for using both the individual ReQoL and the average of the baseline measurement within the corresponding housing unit to avoid cross-linking bias. Group difference in RAS-DS was analysed using the corresponding mixed effect model.

A per-protocol analysis was performed on residents who adhered to the study protocol, defined as participating in at least 70% of the weekly PCRP sessions. Additionally, a post hoc sensitivity analysis (not a priori documented in the SAP) was conducted, excluding municipalities with only one housing unit, as they had been automatically allocated to intervention arm without randomisation. GAS scores were transformed into T-scores with a mean of 50 and a SD of 10, adjusted for an expected intercorrelation between goals of 0.3.^25^ The T-scores are unweighted. All statistical analyses were conducted using R V.4.4.2. For more details about packages and procedures, see online supplemental Appendix 2.

### Findings

**Figure 3:**
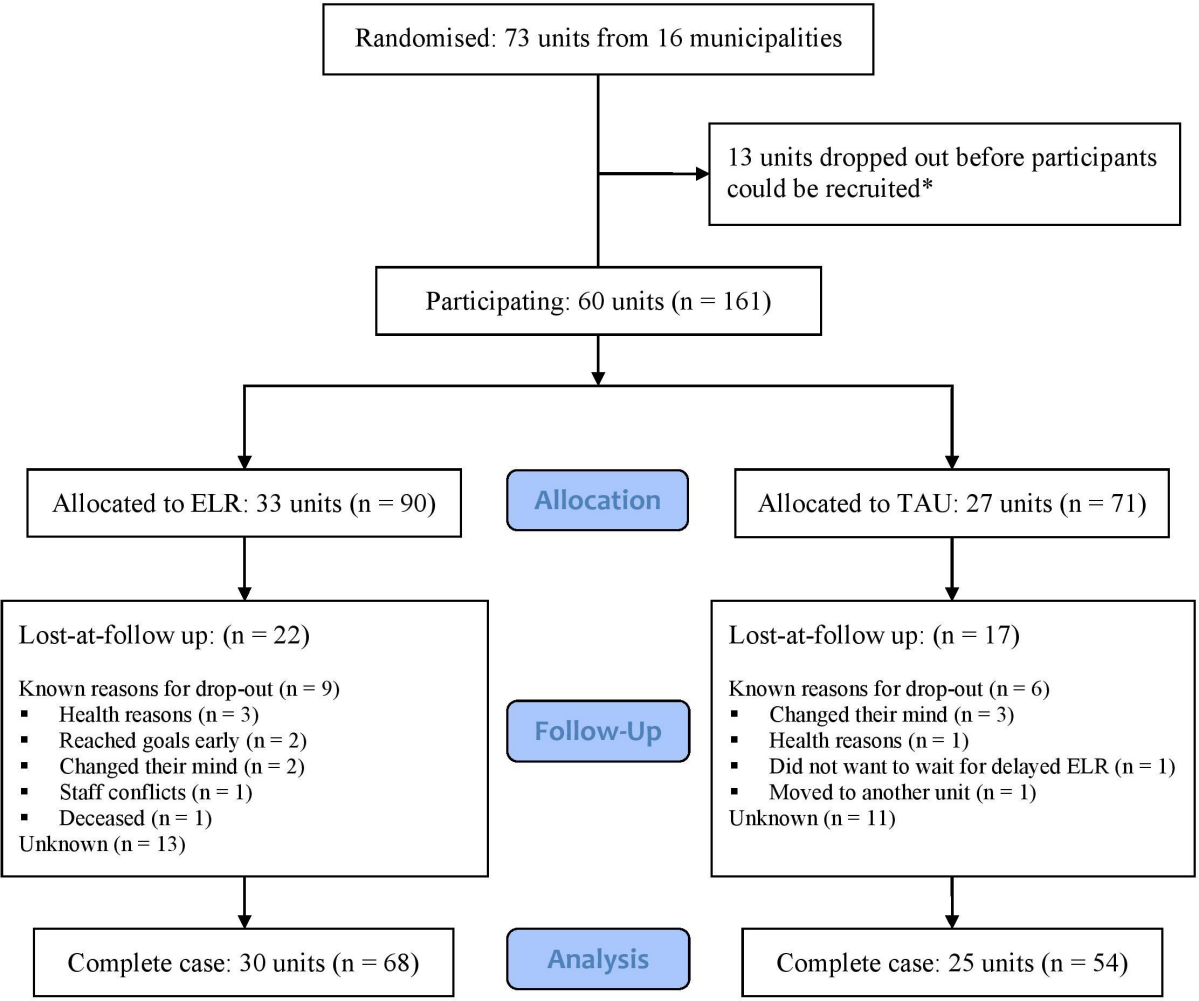
Flowchart. *Two municipalities had to end their participation due to staffing and organisational issues. Some units had to end their participation due to not being able to recruit any eligible participants; these units were not included in the ITT analysis. ELR, everyday life rehabilitation; ITT, intention-to-treat; TAU, treatment-as-usual.

The sample included 60 housing units with 161 participating residents (figure 3). The trial was conducted in four waves, recruiting 73 units from 16 municipalities, with 38 units allocated to ELR and 35 to TAU. Before participant recruitment, two municipalities and 13 units withdrew from the trial (including 5 units from ELR and 8 from TAU). As a result, the final number of units (33 for ELR and 27 for TAU) and participants (90 for ELR and 71 for TAU) differed. The overall attrition rate was 24% in both groups. At the cluster level, the intervention group lost three units (9%), while the control group lost two units (7%) between baseline and the study endpoint.

The median age was 39 years and 54% identified as men. Most (95%) were born in Sweden and had lived in supported accommodation for an average of 7 years. The majority (76%) had upper secondary or equivalent education. Autism (40%) was the most commonly self-reported diagnosis, followed by psychosis (34%). Residents also reported ADHD/ADD (22%; Attention-Deficit/Hyperactivity Disorder), addiction (15%) and bipolar disorder (6%), with 34% selecting ‘other diagnoses’. Multiple diagnoses could be reported. At baseline, 24% had not participated in any organised activities (neither day centre or other regular activities) in the past 2 weeks, and 52% had not met with a friend during that time. Further details are in online supplemental Appendix 2.

**Figure 4:**
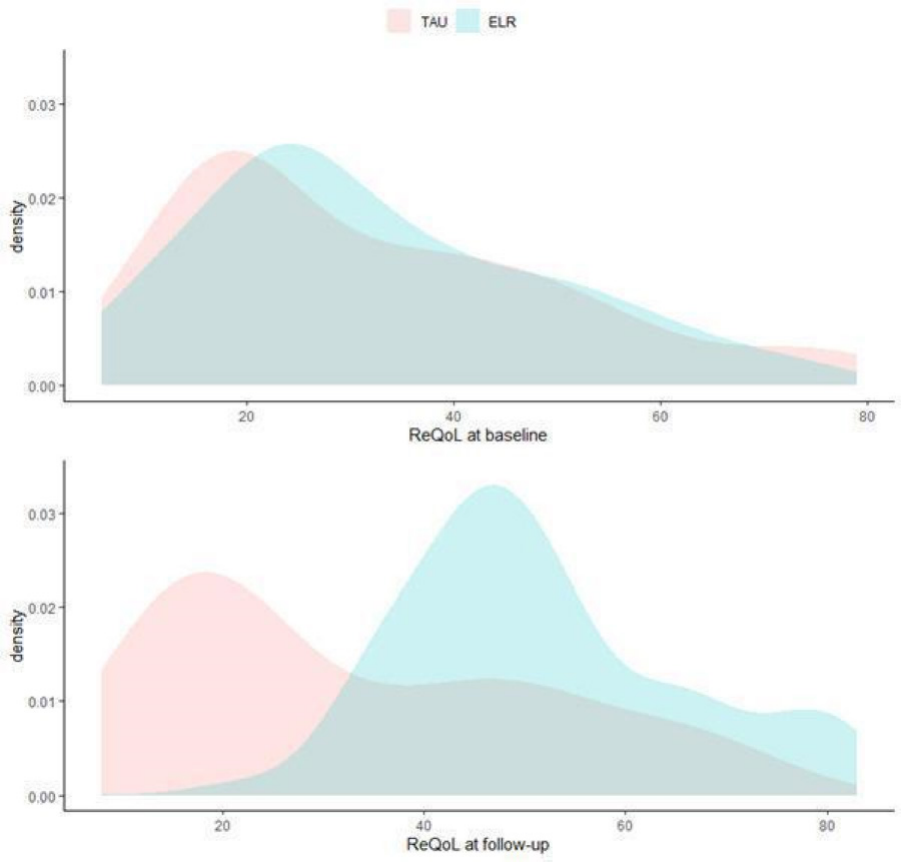
Distribution of ReQoL scores. ELR, everyday life rehabilitation; ReQoL, recovering quality of life; TAU, treatment-as-usual.

The ITT analysis, with 161 individuals from 60 units, demonstrated that the intervention group had significantly higher ReQoL scores compared with TAU at follow-up (mean difference (MD): 20.1, 95% CI: 15.8 to 24.4, p<0.001), see figure 4. The intraclass correlation coefficient (ICC) was 0.28. Additionally, the intervention group had significantly higher RAS-DS scores compared with TAU (MD: 19.0, 95% CI: 13.8 to 24.1, p<0.001, ICC=0.22). The complete-case analysis, based on 122 individuals from 55 units, showed effect sizes similar to those in the ITT analysis for both ReQoL (MD: 20.3, 95% CI: 15.9 to 24.7, p<0.001) and RAS-DS (MD: 22.3, 95% CI: 17.1 to 27.6, p<0.001).

Ancillary analyses were performed on a per-protocol population, consisting of residents who attended at least 70% of their weekly meetings. We collected 73 process protocols from the intervention group. In the per-protocol analysis, based on complete-case data, participants receiving ELR showed significantly higher ReQoL (MD: 20.5, 95% CI: 16.1 to 24.8) and RAS-DS (MD: 22.3, 95% CI: 17.1 to 27.6) scores compared with the control group. No major differences were found between men and women. For more details, see online supplemental Appendix 2. Although the study protocol specified subgroup analyses based on diagnoses, the sample sizes were too small for meaningful analysis. Two municipalities contributed only one unit and were automatically allocated to ELR. Sensitivity analysis excluding them showed similar results (online supplemental Appendix 2).

Residents who received ELR were assessed using GAS. The mean T-score was 53.0, with 89% (n=68) of participants achieving a T-score of 50 or above, indicating that they met or exceeded their expected goals (online supplemental Appendix 2). Only eight individuals reported achieving their goals less than expected. The average number of goals per resident was 1.22. 39 residents did not complete all data collection; reasons were available for 38%. Among those receiving ELR, reasons for dropout included feeling unwell (n=3), achieving goals early (n=2) or changing their mind (n=2). In the TAU group, dropouts were due to changing their mind (n=3), not wanting to wait for the delayed intervention (n=1) or health reasons (n=1). Adverse events included the death of one resident in the intervention group (unrelated to ELR) and another resident who chose to end participation due to conflicts with housing staff.

## Discussion

The main finding of this study was a significant improvement in average ReQoL scores among residents who received ELR, compared with the minimal change observed in the control group. The lower limit of the CI (15.8 points) was well above the 10-point threshold suggested as a clinically important difference,^26^ providing strong evidence of a substantial and meaningful impact on recovering quality of life. Similar results were observed for personal recovery, as measured by the secondary outcome, RAS-DS. Overall, these findings suggest that ELR is an effective intervention for promoting personal recovery in residents with SMI in supported accommodation. At baseline, both groups reported low average ReQoL scores, similar to those observed in clinical populations in the UK.^22^ However, after just 6 months of ELR, the residents’ average scores improved significantly, approaching levels seen in the general population.

These findings provide further evidence that focusing on meaningful everyday activities can lead to a clinically significant improvement in personal recovery and quality of life. Engaging in such activities and participating in real-world community life not only provide structure, other impressions, routine and worthiness but also stimulate connectedness, hope, identity, meaning and empowerment.^15 20 27^ Recovery emphasises the right to access all parts of the community and maintain autonomy, even and especially, while living with a disability or illness. However, individuals with SMI often struggle to be recognised as competent decision-makers.^7 8^ Therefore, signalling hope, prospects, identifying interests and preferences, setting goals and negotiating expectations are important tools for facilitating personal recovery. Emphasising shared decision-making can enhance confidence, foster a sense of ownership and positively influence motivation and long-term recovery outcomes.^28^

A recent systematic review found limited evidence supporting staff training in recovery-oriented practices within supported accommodation.^14^ This suggests that such interventions should not be regarded solely as educational. While training is important, other key components, such as collaborative alliance and recognition of individual autonomy in choosing and engaging in meaningful activities, must also be acknowledged.^29^ Engaging in, exploring and training in real-life activities shifts the focus from staff-led support to active resident involvement in their own recovery. The therapeutic process is complex, influenced by fluctuating moods, conditions and varying levels of motivation.^30^ It requires responsive adaptation, validation, reflection and feedback during progressively challenging activities, as individuals work toward their goals.

Maintaining a high standard of efforts, responsiveness and competence is essential throughout this process. A distinguishing feature of ELR, compared with other interventions, is the active involvement of the occupational therapist, who engages with both residents and staff. They contribute with professional expertise in facilitating meaningful activity and promoting participation, providing a long-term rehabilitative perspective on recovery.^4 15 30^ In a narrative inquiry with participants from this RCT, residents emphasised the importance of meaningful activities as well as the weekly sessions with the occupational therapist.^20^ This support helped them get started and succeed, which positively impacted their self-identity, motivation and life prospects. While the integration of an occupational therapist may be a country-specific component, similar benefits could likely be achieved in other settings as well.

### Limitations

In the sample, the number of allocated housing units and participants differed between the groups. This was partly due to 13 randomised units dropping out before recruitment, meaning no baseline data were available for the ITT analysis. Randomisation occurred at the cluster level before recruitment, with about one-third of residents agreeing to participate, which aligned with the expected recruitment rate. Some units enrolled more or fewer participants than anticipated, affecting the distribution of participants. The occupational therapists were employed by the municipalities and might have served units in both study arms. However, services provided in TAU were brief and sporadic, limiting the risk of contamination.

Municipality recruitment was harder than expected due to crises like COVID-19 and the war in Ukraine (see online supplemental Appendix 2). Despite a large number of clusters, the small cluster sizes present a potential limitation, though consistent with the original power calculation (n=2).^16^ A relatively high dropout rate (24%) was observed, but was comparable to similar trials.^11 13^ Planned subgroup analyses by diagnosis were not feasible due to low data validity and small subgroup sizes. Baseline demographic imbalances between the groups were observed (see online supplemental Appendix 2). No deviations in the randomisation were reported, suggesting that most imbalances are due to random variation. Psychosis was expected to be the most common diagnosis, but autism was most frequently reported, possibly due to under-reporting of severe conditions or multiple diagnoses. In Sweden, eligibility for supported accommodation is not based on diagnosis, but rather the severity of the disability and the need for extensive support in daily life.

The reliance on self-reported diagnoses may reduce diagnostic accuracy, especially given the high proportion of reported conditions usually not included in SMI, such as autism and addiction. While not a major issue for generalising the results within a national context, this could limit the generalisability of the findings in other settings. Future research may consider stronger diagnostic tools to explore how baseline demographics influence the outcome of recovery-oriented interventions. The use of PROMs also lowers evidence certainty but aligns with the intervention’s emphasis on personal recovery, which is not fully captured by objective measures like symptom reduction or service use.

### Clinical implications

This trial provides robust evidence that ELR is an effective intervention for promoting recovering quality of life among individuals with SMI living in supported accommodation. The observed effect size was large and clinically relevant, demonstrating the potential for residents with SMI to progress in recovery and improve their quality of life, even within a relatively short time frame. These findings support the use of person-centred, activity-oriented and recovery-oriented interventions in supported accommodation, emphasising the need for responsive support in goal-oriented weekly activity training, built on collaborative alliance. Further research is needed to identify the most effective strategies for activity-oriented and recovery-oriented interventions and to assess the transferability of ELR to other health systems. In conclusion, this trial provides strong evidence for the effectiveness of ELR. However, to promote the broader adoption of evidence-based interventions in supported accommodation, additional evaluations are needed to assess associated costs and identify strategies to enhance the implementation process.

## Supplementary material

10.1136/bmjment-2025-301757 online supplemental file 1

## Data Availability

Data are available upon reasonable request.
