# Supplementary material for: Effect of Everyday Life Rehabilitation on recovering quality of life in individuals with serious mental illness in supported accommodation: a pragmatic cluster randomised controlled trial
Source: BMJ Ment Health. 2025 Aug 7;28(1):e301757. doi: 10.1136/bmjment-2025-301757 (PMC12336497; doi:10.1136/bmjment-2025-301757)
Supplement: online supplemental file 1 [file bmjment-28-1-s001.docx]

**Appendix 1: Adjustments to the study protocol**

Following the internal pilot, some significant changes were made to the study protocol (Sjöberg et al, submitted 2024). To begin with, to ensure the study had sufficient statistical power, a fourth phase was added including an increased geographical uptake area. This was necessary partly because recruitment proved more challenging than expected, due to several factors, including the COVID-19 pandemic, acute reception of refugees from the war in Ukraine, and the general difficulties municipalities in Sweden face in hiring staff, particularly housing managers.

At the same time, the study's design was redesigned to be able to detect a 10-point difference on the ReQoL scale, an increase from the initially planned 5-point difference. Additionally, after the first wave, all decision-makers within the municipality were provided a preparatory workshop before the start of the study. This approach was implemented to minimize delays in intervention due to organizational unpreparedness, such as lack of routines for intraorganizational collaboration among staff and experience of implementing new methods among upper management.

During the first wave, housing units were informed of their allocation before the baseline measurement so they could prepare for the educational package. In later waves, all units were asked to prepare without knowing their allocation, which allowed blinding to be maintained until the baseline measurement. An updated ethical approval (2023-04148-02) has been granted to accommodate these modifications.

**Sample size recalculations from the internal pilot**

A mixed effects model was fitted with post-measurement ReQoL as dependent variable using the R function lmer from the R package lme4.[1]. The independent fixed effects variables were group, baseline ReQoL, and housing unit was included as a random effect. Using the function powerSim from the R package simR,[2] Monte Carlo simulations with 1000 repetitions were performed to investigate the power of the initially planned sample size. The statistical analysis plan was updated accordingly.[3]

**Appendix 2: Statistical analysis**

**Baseline Characteristics**

| **Characteristic** | **TAU, N = 71^1^** | **ELR, N = 90^1^** |
| --- | --- | --- |
| Age | 41 (32, 52) | 37 (32, 52) |
| **Gender** |  |  |
| Women | 30 (44%) | 42 (47%) |
| Men | 38 (56%) | 48 (53%) |
| **Civil Status** |  |  |
| Partner | 19 (28%) | 9 (10%) |
| Singel | 49 (72%) | 80 (90%) |
| Have children? | 9 (13%) | 18 (20%) |
| Years living in supported housing | 8 (4, 16) | 7 (4, 13) |
| Born in Sweden | 63 (93%) | 87 (97%) |
| **Education level** |  |  |
| Low | 10 (15%) | 17 (19%) |
| Middle | 55 (81%) | 65 (72%) |
| High | 3 (4.4%) | 8 (8.9%) |
| No organized activities in last 2 weeks | 9 (13%) | 29 (33%) |
| No meetings with friends in last 2 weeks | 38 (56%) | 43 (48%) |
| **Mental Health Conditions** |  |  |
| Addiction | 9 (13%) | 14 (16%) |
| ADHD/ADD | 14 (21%) | 21 (24%) |
| Psychosis | 25 (37%) | 28 (31%) |
| Autism/Asperger’s | 26 (38%) | 37 (42%) |
| Bipolar Disorder | 6 (88%) | 4 (4.5%) |
| Personality Disorder | 6 (8.8%) | 5 (5.6%) |
| Other | 22 (32%) | 32 (36%) |
| ^1^ Median (IQR); n (%) | | |

Missing data handled using Multiple Imputations by Chained Equations (MICE) using the R package mice.[4] Mixed effects models were estimated using the lmer function from the R package lme4 [1] and P-values and confidence intervals will be calculated using Satterthwaite's degrees of freedom method, using the lmerTest package.[5]

**Outcomes by arms**

| **Characteristic** | **TAU, N = 71** | **ELR, N = 90** |
| --- | --- | --- |
| Baseline ReQoL scores | 26 (18, 46) | 28 (21, 45) |
| Endpoint ReQoL scores | 26 (18, 49) | 49 (43, 60) |
| Baseline RAS-DS scores | 71 (62, 108) | 78 (63, 101) |
| Endpoint RAS-DS scores | 72 (62, 114) | 99 (87, 111) |
| Median (IQR) | | |

**RAS distributions**

**
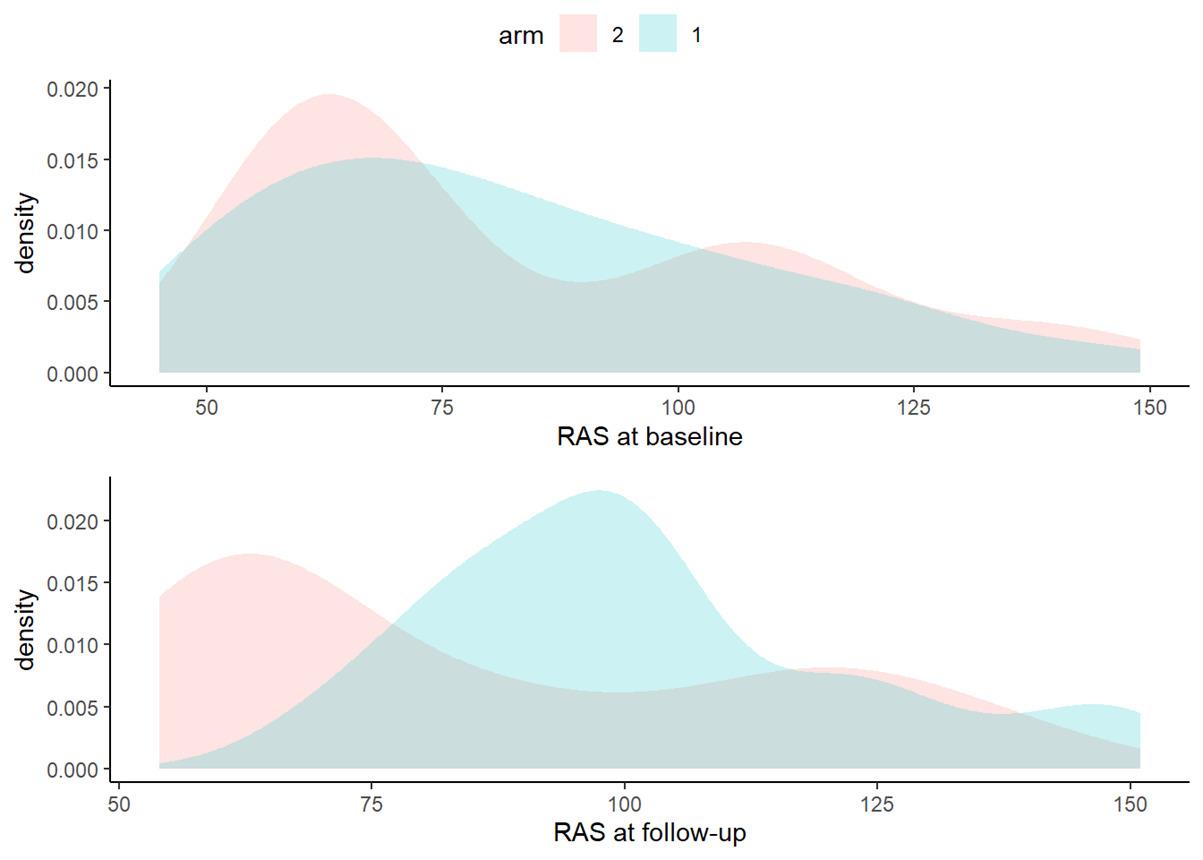
**

**Goal achievement (Goal attainment scale)**

|  | **Number of residents (n)** |
| --- | --- |
| Worse then expeceted (<50) | 8 |
| As expected (=50) | 42 |
| Better then expected (>50) | 26 |

**Ancillary analyses**

| **Analysis** |  | **ReQoL** | **RAS-DS** |
| --- | --- | --- | --- |
| **Complete case-analysis** |  | 20.3 (15.9 to 24.7) | 22.31 (17.1 to 27.6) |
| **Sub-group analysis** | | | |
| Men |  | 19.8 (14.7 to 24.8) | 18.0 (11.1 to 24.9) |
| Women |  | 20.9 (15.3 to 26.5) | 21.3 (14.8 to 27.8) |
| **Per-protocol** **analysis** |  | 20.5 (16.1 to 24.8) | 22.3 (17.1 to 27.6) |

**Sensitivity analysis, excluding the two municipalities with only one housing unit**

|  | **Difference** | **std.error** | **p.value** | **95% CI** |
| --- | --- | --- | --- | --- |
| ReQoL | 20.1 | 2.2 | <0.001 | 15.7 to 24.5 |
| RAS-DS | 19.4 | 2.6 | <0.001 | 14.3 to 24.6 |

**Imputed analyses of group difference**We excluded wave 2 from the analysis due to convergence problems leading to calculational issues due to small sample size (n = 15). The crude mean difference for wave 2 was 20.7 in ReQoL and 18.3 in RAS-DS.

**Imputed analyses of group difference (ELR vs TAU) in ReQoL, estimated from ITT-population and stratified by wave. ***

| **Wave** | **n** | **Difference** | **95% CI** | **P** |
| --- | --- | --- | --- | --- |
| 1 | 33 | 20.3 | 10.5 to 30.1 | <0.001 |
| 3 | 81 | 23.4 | 19.8 to 26.9 | <0.001 |
| 4 | 32 | 9.7 | 0.3 to 19.0 | 0.043 |

**Imputed analyses of group difference (ELR vs TAU) in RAS-DS, estimated from ITT-population and stratified by wave. ***

| **Wave** | **n** | **Difference** | **95% CI** | **P** |
| --- | --- | --- | --- | --- |
| 1 | 33 | 13.2 | 2.0 to 24.4 | 0.023 |
| 3 | 81 | 25.7 | 21.0 to 30.3 | <0.001 |
| 4 | 32 | 9.8 | -9.7 to 29.2 | 0.298 |

**Comparison of descriptive characteristics between participants with complete follow-up measurement of the primary outcome ReQoL and those with missing follow-up.**

Continuous variables are presented as median (IQR), and categorical as n (%). P-value is calculated from Mann-Whitney U-test for continuous variables and chi^2^-square test for categorical variables.

| **Characteristic** | **Complete follow-up** | **Missing follow-up** | **p** |
| --- | --- | --- | --- |
| **Age** | 40 (32, 52) | 39 (32, 51) | 0.94 |
| **Gender** |  |  |  |
| Women | 55 (45%) | 17 (47%) | 0.82 |
| Men | 67 (55%) | 19 (53%) |  |
| **Civil Status** |  |  | 0.023 |
| Partner | 17 (14%) | 11 (31%) |  |
| Singel | 104 (86%) | 25 (69%) |  |
| **Have children?** | 18 (15%) | 9 (25%) | 0.16 |
| **Years living in supported housing** | 8 (4, 15) | 5 (4, 13) | 0.35 |
| **Born in Sweden** | 118 (97%) | 32 (89%) | 0.06 |
| **Education level** |  |  | >0.99 |
| Low | 21 (17%) | 6 (17%) |  |
| Middle | 92 (75%) | 28 (78%) |  |
| High | 9 (7.4%) | 2 (5.6%) |  |
| **No organized activities in last 2 weeks** | 72 (61%) | 21 (58%) | 0.77 |
| **No meetings with friends in last 2 weeks** | 64 (53%) | 17 (47%) | 0.55 |
| **Mental Health Conditions** |  |  |  |
| Addiction | 19 (16%) | 4 (11%) | 0.49 |
| ADHD/ADD | 22 (18%) | 13 (36%) | 0.023 |
| Psychosis | 42 (35%) | 11 (31%) | 0.64 |
| Autism/Asperger’s | 48 (40%) | 15 (42%) | 0.83 |
| Bipolar Disorder | 8 (6.6%) | 2 (5.6%) | 0.82 |
| Personality Disorder | 9 (7.4%) | 2 (5.6%) | 0.70 |
| Other | 42 (35%) | 12 (33%) | 0.88 |

**References**

1 Bates D, Mächler M, Bolker B, *et al.* Fitting Linear Mixed-Effects Models Using lme4. *J Stat Softw*. 2015;67:1–48. doi: 10.18637/jss.v067.i01

2 Green P, MacLeod CJ. SIMR: an R package for power analysis of generalized linear mixed models by simulation. *Methods Ecol Evol*. 2016;7:493–8. doi: 10.1111/2041-210X.12504

3 Lindstrom M. Effect and Cost-effectiveness of the Everyday Life Rehabilitation Intervention: a Pragmatic RCT of Integrated, Recovery-focused Rehabilitation Within Sheltered and Supported Housing Facilities for People With Psychiatric Disabilities. clinicaltrials.gov 2024.

4 Buuren S van, Groothuis-Oudshoorn K. mice: Multivariate Imputation by Chained Equations in R. *J Stat Softw*. 2011;45:1–67. doi: 10.18637/jss.v045.i03

5 Kuznetsova A, Brockhoff PB, Christensen RHB. lmerTest Package: Tests in Linear Mixed Effects Models. *J Stat Softw*. 2017;82:1–26. doi: 10.18637/jss.v082.i13
